# 50 Years of Zweifel Olefination: A Transition-Metal-Free Coupling

**DOI:** 10.1055/s-0036-1589046

**Published:** 2017-07-11

**Authors:** Roly J. Armstrong, Varinder K. Aggarwal

**Affiliations:** School of Chemistry, University of BristolCantock’s Close, Bristol, BS8 1TSUKv.aggarwal@bristol.ac.uk

**Keywords:** Zweifel olefination, coupling, boronic esters, alkenes, transition­-metal-free, enantiospecific

## Abstract

The Zweifel olefination is a powerful method for the stereoselective synthesis of alkenes. The reaction proceeds in the absence of a transition-metal catalyst, instead taking place by iodination of vinyl boronate complexes. Pioneering studies into this reaction were reported in 1967 and this short review summarizes developments in the field over the past 50 years. An account of how the Zweifel olefination was modified to enable the coupling of robust and air-stable boronic esters is presented followed by a summary of current state of the art developments in the field, including stereodivergent olefination and alkynylation. Finally, selected applications of the Zweifel olefination in target-oriented synthesis are reviewed.

1 Introduction

2.1 Zweifel Olefination of Vinyl Boranes

2.2 Zweifel Olefination of Vinyl Borinic Esters

2.3 Extension to Boronic Esters

3.1 Introduction of an Unsubstituted Vinyl Group

3.2 Coupling of α-Substituted Vinyl Partners

3.3 *Syn* Elimination

4 Zweifel Olefination in Natural Product Synthesis

5 Conclusions and Outlook

## 1 Introduction

The stereocontrolled synthesis of alkenes is a topic that has attracted a great deal of attention owing to the prevalence of this motif in natural products, pharmaceutical agents and materials. 1 Of the many olefination methods that exist, the Suzuki–Miyaura coupling represents a highly convergent method to assemble alkenes (Scheme 1, a ). 2 However, although the coupling of vinyl halides with primary and sp ^2^ boronates takes place effectively, the coupling of secondary and tertiary (chiral) boronates remains problematic. 3 Furthermore, the high cost and toxicity of the palladium complexes required to catalyze these processes also detract from the appeal of this methodology. 4

**Varinder K. Aggarwal:**
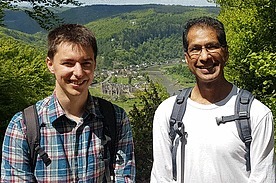
(right) studied chemistry at Cambridge University and received his Ph.D. in 1986 under the guidance of Dr Stuart Warren­. After postdoctoral studies (1986–1988) under Professor Gilbert Stork, Columbia University, he returned to the UK as a Lecturer at Bath University. In 1991, he moved to Sheffield University, where he was promoted to Professor in 1997. In 2000, he moved to Bristol University where he holds the Chair in Synthetic Chemistry. He was elected Fellow of the Royal Society in 2012. **Roly J. Armstrong** (left) graduated with an MSci in Natural Sciences from Pembroke College, Cambridge (2011) spending his final year working in the laboratory of Professor Steven Ley. He subsequently moved to Merton College, Oxford to carry out a DPhil under the supervision of Professor Martin Smith (2011–2015) working on asymmetric counterion-directed catalysis. In October 2015, he joined the group of Professor Varinder Aggarwal at the University of Bristol as a postdoctoral research associate.

The Zweifel olefination represents a powerful alternative to the Suzuki–Miyaura reaction, enabling the coupling of vinyl metals with enantioenriched secondary and tertiary boronic esters with complete enantiospecificity (Scheme 1, b ). 5 The reaction is mediated by iodine and base and proceeds with no requirement for a transition-metal catalyst. This process is based upon pioneering studies reported in 1967 by Zweifel and co-workers on the iodination of vinyl boranes. This short review summarizes the key contributions made over the last 50 years that have enabled this transformation to evolve into an efficient and atom-economical method for the coupling of boronic esters. Recent contributions to the field are described including the development of Grignard-based vinylation, stereodivergent olefination and alkynylation processes. Finally, selected examples of Zweifel olefination in target-oriented synthesis are reviewed to highlight the utility of this methodology.


**Scheme 1** Olefination of boronic esters


### 2.1 Zweifel Olefination of Vinyl Boranes

In 1967, Zweifel and co-workers reported that vinyl boranes­ **1** , obtained by hydroboration of the corresponding alkynes, could be treated with sodium hydroxide and iodine­, resulting in the formation of alkene products **2** (Scheme 2 ). 6 Intriguingly, although the intermediate vinyl boranes were formed with high *E* -selectivity, after addition of iodine, *Z* -alkenes were produced. A reaction with a dia­stereomerically pure secondary borane afforded the coupled product, **2d** , as a single *anti* diastereoisomer, indicating that the process proceeds with retention of configuration. 7 Mechanistically, this reaction is thought to proceed by activation of the π bond with iodine along with complexation of sodium hydroxide to form a zwitterionic iodonium intermediate **3** . This species is poised to undergo a stereospecific 1,2-metalate rearrangement resulting in the formation of a β-iodoborinic acid **4** . In the presence of sodium hydroxide, this intermediate then undergoes *anti* elimination to afford the resulting *Z* -alkene product. 8


**Scheme 2** Zweifel olefination: iodination of vinyl boranes


Because vinyl borane intermediates could only be accessed by hydroboration of alkynes, the iodine-mediated Zweifel coupling was initially limited to the synthesis of *Z* -alkenes. 9 However, Zweifel and co-workers subsequently reported an elegant strategy for the complementary synthesis of *E* -alkenes (Scheme 3 ). 10 This transformation was achieved by reacting dialkyl vinyl borane **5** with cyanogen bromide under base-free conditions. Following stereospecific bromination, a boranecarbonitrile intermediate **8** , was formed, a species that was sufficiently electrophilic to undergo *syn* elimination. A variety of boranes underwent this transformation, forming alkenes **6a** – **c** in high yields and with very high levels of *E* -selectivity. Chiral non-racemic boranes could be transformed with complete stereospecificity.


**Scheme 3** Synthesis of *E* -alkenes using cyanogen bromide


A related *syn* elimination process was reported by Levy and co-workers (Scheme 4 ). 11 In this case, a vinyl lithium reagent was prepared by lithium–halogen exchange and then combined with a symmetrical trialkylborane resulting in formation of boronate complex **9** . Treatment of this intermediate with iodine resulted in stereospecific iodination to produce β-iodoborane **10** . The enhanced electrophilicity of this species (compared to β-iodoborinic acids such as **4** ) enabled a *syn* elimination to occur, generating the corresponding trisubstituted alkene **11** with high levels of stereocontrol. Although the substrate scope of the process is wide, the method was limited to the use of symmetrical trialkyl boranes.


**Scheme 4** Olefination of symmetrical trialkylboranes



**Scheme 5** Alkynylation of boranes


Brown and co-workers demonstrated that the Zweifel olefination can also be applied to the synthesis of alkynes (Scheme 5 ). 12 In this case, monosubstituted alkynes were deprotonated to form lithium acetylides, which were reacted with trialkylboranes to form alkynylboronate complexes **12** . Addition of iodine triggered a 1,2-metallate rearrangement to generate β-iodoboranes **13** , which spontaneously underwent elimination to form alkyne products. This process represents a convenient alternative to the alkylation of lithium acetylides with alkyl halides and has been successfully employed in total synthesis. 13

### 2.2 Zweifel Olefination of Vinyl Borinic Esters


**Scheme 6** Diene synthesis by Zweifel olefination of boranes or borinic esters


The transformations described in the previous section suffer from an inherent limitation in that only one of the alkyl groups present in the borane starting materials is incorporated into the alkene product. This is particularly wasteful when the borane is challenging to access or expensive. One solution to this problem would be to employ a mixed borane in which one (or two) of the boron-bound groups demonstrates a low migratory aptitude (e.g., thexyl). 14 However, in practice, determining which group will migrate has proved to be non-trivial and highly substrate-dependent. For example, Zweifel and co-workers showed that treatment of divinylalkylborane **15** (obtained by double hydroboration of 1-hexyne with thexylborane) with iodine resulted in competitive migration of both the sp ^2^ and thexyl groups leading to a mixture of the desired product **16** along with **17** (Scheme 6 ). 15 They overcame this problem by treating the intermediate divinylalkylborane **15** with trimethylamine oxide, resulting in selective oxidation of the B–C _thexyl_ bond to afford borinic ester **18** . Due to the low migratory aptitude of an alkoxy ligand on boron, 16 addition of iodine and sodium hydroxide now led to selective formation of *Z* , *E* -diene **16** . Although this allowed control over which group migrated, the method was limited to the synthesis of symmetrical dienes.

A more general approach to the iodination of vinyl borinic esters was later reported by Brown and co-workers (Scheme 7 ). 17 In this case, non-symmetrical vinyl borinic esters **20** were obtained by hydroboration of alkynes with alkylbromoboranes followed by methanolysis of the resulting bromoborane intermediates **19** . Addition of sodium methoxide­ and iodine led to alkene products **21a** – **d** in good yields and very high levels of *Z* -selectivity.


**Scheme 7** Synthesis of *Z* -alkenes from vinyl borinic esters


### 2.3 Extension to Boronic Esters

Although the use of borinic esters significantly expanded the potential of the Zweifel olefination, there were still significant problems with this approach, most notably associated with the high air sensitivity of the borane starting materials. In contrast to boranes, boronic esters are air- and moisture-stable materials which can be readily prepared via a wide range of methods. 18 Evans and Matteson independently recognized the potential of boronic esters as substrates for Zweifel olefination communicating independent studies almost simultaneously. 19 20

Matteson’s coupling process began with the synthesis of a vinyl boronate complex **23** by addition of an organolithium reagent to a vinyl boronic ester **22** (Scheme 8 ). 19 This intermediate was treated with iodine and sodium hydroxide, resulting in iodination followed by 1,2-metallate rearrangement to form a β-iodoboronic ester which underwent *anti* elimination to form the corresponding *Z* -alkene. This reaction could be carried out with alkyl or aryl lithium reagents and the coupled products **24a** and **24b** were formed in moderate to good yields.


**Scheme 8** Zweifel olefination of vinyl boronic esters


Evans and co-workers’ strategy also began with formation of a vinyl boronate complex (Scheme 9 ). 20 In contrast to Matteson’s approach, this intermediate was accessed by reacting *E* -vinyl lithium reagent **26a** (prepared by lithium–halogen exchange) with secondary alkyl boronic ester **25** . Treatment of the resulting vinyl boronate complex **27a** with iodine and sodium methoxide resulted in formation of alkene **28a** in 75 % yield (>96:4 *Z* / *E* ). When a *Z* -vinyl lithium precursor **26b** was employed, alkene **28b** was obtained in 58 % yield with very high *E* -selectivity. The flexibility derived from the ability to form identical vinyl boronate complexes by either reacting a vinyl boronic ester with an organolithium or a vinyl lithium with a boronic ester is a particularly appealing feature of the Zweifel olefination.


**Scheme 9** Zweifel olefination of vinyl lithiums with boronic esters


Brown and co-workers subsequently extended this methodology to enable the synthesis of trisubstituted alkenes (Scheme 10 ). 17c 21 By reacting various trisubstituted vinyl boronic esters ( **29** ) with organolithium nucleophiles, a range of products was prepared in good to excellent yields. Notably, heteroaromatic groups could be introduced (in **31b** ) and alkyl Grignard reagents could be used in place of organolithium reagents (in **31d** ).


**Scheme 10** Zweifel olefination of trisubstituted vinyl boronic esters


The methods shown in Schemes 8–10 represented a significant advance upon the early work on the Zweifel olefination of boranes and borinic esters. However, at the time the potential of the method was not fully realized owing to the paucity of methods available for the preparation of boronic esters. Consequently, only a handful of studies involving Zweifel olefination were published over the following three decades. 22 In recent years, the huge increase in methods available for the enantioselective synthesis of boronic esters has led to a renaissance in chemistry based upon the Zweifel olefination. Several new studies into the process have been reported along with elegant reports employing Zweifel olefination in total synthesis. These results are described in the following sections.

### 3.1 Introduction of an Unsubstituted Vinyl Group

The introduction of a vinyl group into a target molecule is commonly required in synthesis owing to the prevalence of this motif in natural products and as a valuable handle for further functionalization. The first report describing the introduction of an unsubstituted vinyl group by Zweifel olefination was published by Aggarwal and co-workers in their stereocontrolled synthesis of (+)-faranal (Scheme 11 ). 23 In this process, vinyl lithium was prepared in situ from tetravinyltin by tin–lithium exchange and was then reacted with enantioenriched secondary boronic ester **32** . The resulting vinyl boronate complex was treated with iodine and sodium methoxide, thus promoting 1,2-metallate rearrangement and elimination affording alkene **33** . This key intermediate was directly subjected to hydroboration and oxidation to provide alcohol **34** in 69 % yield with very high diastereoselectivity. Oxidation with PCC completed the synthesis of (+)-faranal in 76 % yield.


**Scheme 11** Introduction of an unsubstituted vinyl group with vinyl lithium: stereoselective synthesis of (+)-faranal; R = (CH _2_ ) _2_ CHCMeEt; pin = pinacolato


It was subsequently shown that the vinyl lithium approach could be also be applied to the enantiospecific coupling of trialkyl tertiary boronic esters (Scheme 12, a ) 24 and benzylic tertiary boronic esters 25 (Scheme 12, b ). It is noteworthy that in these cases despite the sterically congested nature of the boronic ester starting materials, the coupled products were obtained in excellent yields. The double vinylation of primary–tertiary 1,2-bis(boronic esters) has also been achieved using this approach (Scheme 12, c ). 26 Using four equivalents of vinyl lithium, diene **37** was obtained in 77 % yield.


**Scheme 12** Applications of Zweifel olefination with vinyl lithium (prepared from tetravinyltin); PMP = *p* -methoxyphenyl; e.s. = enantiospecificity


Vinylation under Zweifel conditions represents a powerful strategy for the synthesis of alkenes. However, the necessity of preparing vinyl lithium in situ from the corresponding toxic stannane or volatile vinyl bromide detracts from the appeal of the process. In contrast, stable THF solutions of vinylmagnesium chloride or bromide are commercially available. 27 Aggarwal and co-workers have studied the Zweifel olefination of tertiary boronic ester **38** with vinylmagnesium bromide in THF. 25 Monitoring the reaction by ^11^ B NMR spectroscopy revealed that with one equivalent of vinylmagnesium bromide, the expected vinyl boronate complex **39** was not observed and instead a mixture of unreacted boronic ester **38** and trivinyl boronate complex **40** was formed (Scheme 13 ). The latter species originates from over-addition of vinylmagnesium bromide promoted by the high Lewis acidity of the Mg ^2+^ counterion. Upon addition of an excess of vinylmagnesium bromide (4 eq.), trivinyl boronate complex **40** was obtained exclusively, and after addition of I _2_ followed by NaOMe, the coupled product **41a** was obtained in good yield. These conditions were successfully applied to the synthesis of a series of benzylic tertiary substrates **41a** – **d** . The reaction is ineffective at forming very hindered alkenes such as **36** , although this product could be synthesized efficiently with vinyl lithium.


**Scheme 13** Zweifel olefination of tertiary boronic esters with vinylmagnesium bromide in THF



**Scheme 14** Zweifel olefination of boronic esters with vinylmagnesium chloride in THF/DMSO; R = (CH _2_ ) _2_ PMP


Very recently, an improved procedure for coupling unhindered boronic esters with vinylmagnesium chloride has been reported (Scheme 14 ). 28 As with tertiary boronic esters, it was observed that addition of vinylmagnesium chloride to a THF solution of secondary boronic ester **42** resulted in over-addition to form trivinyl boronate complex **44** . However, if the reaction was carried out in a 1:1 THF/DMSO mixture, 29 over-addition was completely suppressed and only mono-vinyl boronate complex **43** was obtained. After addition of iodine and sodium methoxide, the coupled product **45a** was obtained in 89 % yield. This process proceeds effectively with a range of primary, secondary and aromatic boronic esters. Notably, the use of the mild Grignard reagent allows chemoselective coupling to occur in the presence of reactive functional groups such as carbamates (in **45b** ) and ethyl esters (in **45d** ). Although good yields of product were obtained with unhindered tertiary boronic esters (in **45e** ), in general, the Zweifel vinylation of tertiary boronic esters is best achieved either with four equivalents of vinylmagnesium halide in THF or with vinyl lithium.

In summary, there are currently three methods available to introduce an unsubstituted vinyl group by Zweifel olefination (Scheme 15 ). For aromatic, primary and unhindered secondary boronic esters, the desired boronate complex can be formed efficiently using 1.2 equivalents of vinylmagnesium halide in 1:1 THF/DMSO. For the majority of tertiary boronic esters it is recommended to employ four equivalents of vinylmagnesium halide in THF (to form the trivinyl boronate complex), although with extremely hindered tertiary boronic esters, the best results are obtained with vinyl lithium.


**Scheme 15** Summary of the best methods for boronate complex formation for the Zweifel vinylation of various boronic esters; R = alkyl group


### 3.2 Coupling of α-Substituted Vinyl Partners

In addition to the synthesis of alkyl-substituted alkenes, the Zweifel olefination has also been applied to the coupling of vinyl partners α-substituted with a heteroatom. The coupling of lithiated ethyl vinyl ether **46** (readily prepared by deprotonation of ethoxyethene with *^t^* BuLi) with a tertiary boronic ester proceeded smoothly to provide enol ether **47** , which was hydrolyzed under mild conditions to form **48** (Scheme 16, a ). 25 30 This process represents a novel method for the conversion of boronic esters into ketones. This methodology has also been extended to the enantiospecific synthesis of vinyl sulfides (Scheme 16, b ). 28


**Scheme 16** Synthesis of ketones and vinyl sulfides by Zweifel olefination


A related strategy for the alkynylation of boronic esters has recently been reported by Aggarwal and co-workers (Scheme 17 ). 31 In contrast to the successful alkynylation reactions of trialkyl boranes discussed previously (Scheme 5 ), 12 13 boronic esters undergo reversible boronate complex formation with lithium acetylides. This means that addition of electrophiles does not result in coupling, but instead leads to direct trapping of the acetylide and recovery of the boronic ester. A solution to this problem was developed in which vinyl bromides or carbamates were lithiated at the α-position with LDA and then reacted with boronic esters in a Zweifel olefination. Treatment of the resulting vinyl bromides or carbamates with base (TBAF for bromides and *^t^* BuLi or LDA for carbamates) triggered elimination to form the corresponding alkynes **50** . Coupling of a wide range of secondary and tertiary boronic esters was achieved in excellent yields with complete enantiospecificity.

In 2014, an interesting intramolecular variant of the Zweifel olefination for the construction of four-membered ring products was reported (Scheme 18 ). 32 In this process, **51** , which possesses both a boronic ester and a vinyl bromide, was treated with *tert* -butyllithium resulting in chemoselective lithium–halogen exchange followed by spontaneous cyclization to form cyclic vinyl boronate complex **52** . Upon treatment with iodine and methanol this species underwent stereospecific ring contraction to provide β-iodo­boronic ester **53** . Elimination of this intermediate gave exocyclic alkene **54** in 63 % yield. It is particularly noteworthy that this challenging Zweifel olefination occurs in good yield despite the highly strained nature of the exomethylene cyclobutene product.


**Scheme 17** Alkynylation of enantioenriched boronic esters; Cb = C(O)N *^i^* Pr _2_



**Scheme 18** Construction of an exomethylene cyclobutene by an intramolecular Zweifel olefination; Ar = 2-MeO-4-MeC _6_ H _3_


### 3.3 *Syn* Elimination

Aggarwal and co-workers have reported a method for the synthesis of allylsilanes through a lithiation–borylation­–Zweifel olefination strategy (Scheme 19 ). 33 In this process, silaboronate **56** was homologated with configurationally stable lithium carbenoids **55** to provide α-silylboronic esters **57** , which were then subjected to Zweifel olefination to obtain allylsilane products **58** . Notably, it was necessary to carry out the Zweifel olefination without sodium methoxide owing to the instability of the allylsilane products under basic conditions. The substrate scope of the process was wide and a range of allylsilanes was prepared in high yields and with excellent levels of enantioselectivity. Interestingly, with a hindered α-silylboronic ester, *E* -crotylsilane **58d** was obtained as a single geometrical isomer, but *Z* -crotylsilane **58c** was formed in slightly reduced selectivity (95:5 *Z* / *E* ).


**Scheme 19** Synthesis of allyl- and crotylsilanes via a lithiation–borylation–Zweifel olefination strategy; *Si* = SiPhMe _2_ ; (–)-sp = (–)-sparteine


To rationalize the reduced selectivity observed in the formation of *Z* -crotylsilane **58c** , it was postulated that as the boronic ester becomes more hindered, the transition state for *anti* elimination becomes disfavored due to a steric clash between the bulky R ^1^ and R ^2^ substituents (Scheme 20 ). This allows the usually less favorable *syn* elimination pathway to compete, resulting in the formation of small amounts of the *E* -isomer.


**Scheme 20** Rationalization for reduced *Z* / *E* selectivity with bulky boronic­ esters


Similar behavior has been observed in the Zweifel olefination of hindered secondary boronic esters with alkenyllithiums (Scheme 21 ). 34 As the boronic ester becomes more sterically encumbered (for example, benzylic or β-branched), increasing formation of the *E* -isomer was observed, up to 90:10 *Z* / *E* in the case of menthol-derived alkene **60c** .


**Scheme 21** Reduced *Z* / *E* selectivity with bulky boronic esters


In these cases, Aggarwal and co-workers have shown that iodine can be replaced with PhSeCl resulting in the formation of β-selenoboronic esters (Scheme 22 ). 35 Because the selenide is a poorer leaving group than the corresponding iodide, treatment of these intermediates with sodium methoxide led exclusively to *anti* elimination providing the coupled products **60a** – **c** as a single *Z* -isomer in all cases. 34


**Scheme 22** Highly *Z* -selective olefination of sterically hindered boronic esters



**Scheme 23** Stereodivergent olefination of boronic esters


It was also demonstrated that β-selenoboronic esters (obtained by selenation of vinyl boronate complexes) could be directly treated with *m* -CPBA resulting in chemoselective oxidation of the selenide to give the corresponding selenoxide (Scheme 23, a ). 34 A novel *syn* elimination then occurred in which the selenoxide oxygen atom attacked a boron atom instead of a hydrogen atom, providing *E* -alkenes with high selectivity. In conjunction with the Zweifel olefination (or its PhSeCl-mediated analogue) this represents a stereodivergent method where either isomer of a coupled product can be obtained from a single isomer of vinyl bromide starting material (Scheme 23, b ). The substrate scope of both processes is broad and a range of di- and trisubstituted alkenes was prepared including **61c** which represents the C9–C17 fragment of the natural product discodermolide.

In some cases, the ability to carry out *syn* elimination of β-iodoboronic esters is also desirable. For example, very recently Aggarwal and co-workers reported a coupling of cyclic vinyl lithium reagents with boronic esters (Scheme 24 ). 28 In this case, the cyclic β-iodoboronic ester intermediates **63** cannot undergo bond rotation and therefore must undergo a challenging *syn* elimination. It was found that this elimination could be promoted by adding an excess of sodium methoxide (up to 20 eq.). Using this methodology, a range of five- and six-membered cycloalkene products **64** were prepared in high yields and with complete stereospecificity, including glycal **64b** and abiraterone derivatives such as **64c** .


**Scheme 24** Synthesis of cycloalkenes via a challenging *syn* elimination


Since the pioneering studies on Zweifel olefination reported by Evans and Matteson, the method has been significantly developed such that a wide range of functionalized alkene products can now be obtained. The final section of this short review showcases selected examples where Zweifel olefination has been used in complex molecule synthesis. 36

## 4 Zweifel Olefination in Natural Product Synthesis

Aggarwal and co-workers recently reported an 11-step total synthesis of the alkaloid (–)-stemaphylline employing a tandem lithiation–borylation–Zweifel olefination strategy (Scheme 25 ). 37 Pyrrolidine-derived boronic ester **65** was homologated with a lithium carbenoid to afford boronic ester **66** in 58 % yield and 96:4 d.r. A subsequent Zweifel olefination with vinyl lithium (synthesized in situ from tetra­vinyltin) gave alkene **67** in 71 % yield. Notably, these two steps could be combined into a one-pot operation, directly providing **67** in 70 % yield. The alkene was later employed in a ring-closing-metathesis–reduction sequence to form the core 5-7 ring system of (–)-stemaphylline.


**Scheme 25** Stereocontrolled synthesis of (–)-stemaphylline; *Si* = TBDPS; TIB = 2,4,6-triisoproylbenzoyl


A recent formal synthesis of the complex terpenoid natural product solanoeclepin A has been reported by Hiemstra and co-workers (Scheme 26 ). 38 A key step in this synthesis was the vinylation of the bridgehead tertiary boronic ester in **68** . Formation of the trivinyl boronate complex with excess vinylmagnesium bromide in THF followed by addition of iodine and sodium methoxide produced alkene **69** , which was employed without purification in a subsequent sequence of oxidative cleavage and Horner–Wadsworth–Emmons olefination to form **70** in a yield of 67 % over four steps.


**Scheme 26** Formal synthesis of solanoeclepin A



**Scheme 27** Enantioselective synthesis of debromohamigeran E


Morken and Blaisdell have reported an elegant stereoselective synthesis of debromohamigeran E that employs a Zweifel coupling of an α-substituted vinyl lithium (Scheme 27 ). 39 Cyclopentyl boronic ester **72** was prepared from 1,2-bis(boronic ester) **71** in 42% yield by a highly selective hydroxy-directed Suzuki–Miyaura coupling. This intermediate was then subjected to Zweifel coupling with isopropenyllithium (synthesized by Li–Br exchange) to form **73** in 93 % yield. Completion of the synthesis of debromohamigeran E required four further steps including hydrogenation of the alkene to an isopropyl group.

A short enantioselective total synthesis tatanan A was reported by Aggarwal and co-workers, which employs a stereospecific alkynylation reaction (Scheme 28 ). 40 Boronic ester **74** (synthesized by a diastereoselective Matteson homologation) was subjected to Zweifel olefination with lithiated vinyl carbamate. Treatment of the resulting vinyl carbamate **75** with LDA resulted in elimination to form alkyne **76** in 97 % yield with complete diastereospecificity. This alkyne was converted into the trisubstituted alkene of tatanan A in two further steps.


**Scheme 28** Total synthesis of tatanan A; Ar = 2,4,5-trimethoxyphenyl; d.s. = diastereospecifity


A collaborative study on the synthesis of ladderane natural products was recently published by the groups of Boxer, Gonzalez-Martinez and Burns (Scheme 29 ). 41 A key intermediate in these studies was the unusual lipid tail [5]-ladderanoic acid. This compound was prepared from *meso* -alkene **77** by a sequence involving copper-catalyzed desymmetrizing hydroboration (95 % yield, 90 % ee) followed by Zweifel olefination with vinyl lithium reagent **79** (3:1 *E* / *Z* ). It was found that carrying out the Zweifel olefination with *N* -bromosuccinimide rather than iodine was critical to achieve efficient coupling. Following silyl deprotection, the coupled product **80** was obtained in 88 % yield as an inconsequential mixture of *Z* / *E* isomers. Hydrogenation of the alkene followed by Jones oxidation of the primary alcohol completed the first catalytic enantioselective synthesis of [5]-ladderanoic acid.


**Scheme 29** Zweifel olefination in the synthesis of [5]-ladderanoic acid


Negishi and co-workers have employed a Zweifel olefination in the synthesis of the side chain of (+)-scyphostatin (Scheme 30 ). 42 In this case, a boronate complex was formed between vinyl boronic ester **81** (prepared in 7 steps from allyl alcohol) and methyllithium. After addition of iodine and NaOH followed by silyl deprotection, trisubstituted alkene **82** was obtained in 76 % yield. The very high stereoselectivity obtained in this reaction (>98:2 *E* / *Z* ) is particularly noteworthy and represents a significant improvement upon previous synthetic approaches toward this fragment.


**Scheme 30** Construction of the side chain of (+)-scyphostatin


Hoveyda and co-workers have employed a similar strategy to synthesize the antitumor agent herboxidiene (Scheme 31 ). 43 In this case, *Z* -vinyl boronic ester **83** was prepared as a single stereoisomer by a Cu-catalyzed borylation–allylic substitution reaction. Boronic ester **83** was then converted into trisubstituted alkene **84** in a Zweifel olefination with methyllithium. The resulting alkene was obtained as a single *E* -isomer in 70 % yield and could be converted into herboxidiene in five steps.


**Scheme 31** Total synthesis of herboxidiene; BOM = benzyloxymethyl


A stereocontrolled synthesis of (–)-filiformin has been reported by Aggarwal and co-workers involving an intramolecular Zweifel olefination (Scheme 32 ). 32 Intermediate **85** (synthesized in high stereoselectivity by lithiation–borylation­) was converted into cyclic boronate complex **86** by in situ lithium–halogen exchange. Addition of iodine and methanol brought about the desired ring contraction to provide exocyclic alkene **87** in 97 % yield. Deprotection of the phenolic ether followed by acid-promoted cyclization and bromination completed the synthesis of (–)-filiformin.


**Scheme 32** Synthesis of (–)-filiformin via an intramolecular Zweifel olefination


## 5 Conclusions and Outlook

Fifty years have passed since the first report by Zweifel and co-workers on the iodine-mediated olefination of vinyl boranes. Since then, this process has evolved into a robust and practical method for the enantiospecific coupling of boronic esters with vinyl metals. Recent contributions have significantly expanded the generality of the process, enabling the efficient coupling of a wide range of different alkenyl partners and allowing increasingly precise control over the stereochemical outcome of the transformation. Rapid progress in enantioselective boronic ester synthesis combined with the extensive applications of chiral alkenes bode well for the continued development and application of the Zweifel olefination in synthesis.
